# A Novel Genetic Algorithm-Based Optimization Framework for the Improvement of Near-Infrared Quantitative Calibration Models

**DOI:** 10.1155/2020/7686724

**Published:** 2020-07-10

**Authors:** Quanxi Feng, Huazhou Chen, Hai Xie, Ken Cai, Bin Lin, Lili Xu

**Affiliations:** ^1^College of Science, Guilin University of Technology, Guilin 541004, China; ^2^Center for Data Analysis and Algorithm Technology, Guilin University of Technology, Guilin 541004, China; ^3^College of Automation, Zhongkai University of Agriculture and Engineering, Guangzhou 510225, China; ^4^College of Marine Sciences, Beibu Gulf University, Qinzhou 535011, China

## Abstract

The global fishmeal production is used for animal feed, and protein is the main component that provides nutrition to animals. In order to monitor and control the nutrition supply to animal husbandry, near-infrared (NIR) technology was utilized for rapid detection of protein contents in fishmeal samples. The aim of the NIR quantitative calibration is to enhance the model prediction ability, where the study of chemometric algorithms is inevitably on demand. In this work, a novel optimization framework of GSMW-LPC-GA was constructed for NIR calibration. In the framework, some informative NIR wavebands were selected by grid search moving window (GSMW) strategy, and then the variables/wavelengths in the waveband were transformed to latent principal components (LPCs) as the inputs for genetic algorithm (GA) optimization. GA operates in iterations as implementation for the secondary optimization of NIR wavebands. In steps of the variable's population evolution, the parametric scaling mode was investigated for the optimal determination of the crossover probability and the mutation operator. With the GSMW-LPC-GA framework, the NIR prediction effect on fishmeal protein was experimentally better than the effect by simply adopting the moving window calibration model. The results demonstrate that the proposed framework is suitable for NIR quantitative determination of fishmeal protein. GA was eventually regarded as an implementable method providing an efficient strategy for improving the performance of NIR calibration models. The framework is expected to provide an efficient strategy for analyzing some unknown changes and influence of various fertilizers.

## 1. Introduction

Fishmeal is a kind of popular animal food that provides high contents of protein and amino and fatty acids, which are essential nutrients in many formulated diets [1, 2]. Hence, fishmeal industry is widely spread across the world [3]. Approximately 65% of the annual global fishmeal production is used for animal feed, including poultry, swine, and even human foods [4]. In fishmeal manufacture, the nutrition supply is mainly summarized as the variability of protein, which generally accounts for 52–75% of the different fish species. Uniform protein composition is induced and produced from raw materials so that the detection of protein content is essential to guarantee the nutrient quality of fishmeal [5]. The conventional method to determine the protein content is the Kjeldahl method. However, the Kjeldahl method requires chemical operation skills, needs chemical reagents, and is time-consuming [6]. Therefore, the study of rapid detection technologies is inevitable for precise determination of protein content in the process control of fishmeal production.

Near-infrared (NIR) spectroscopy is a rapid detection technology that is able to record the response of the chemical functional groups such as C-H, O-H, and N-H bands, which are the primary chemical compositions of fishmeal protein [7, 8]. NIR spectroscopy shows its advantages in easy and fast application with no destruction to samples and minimal requirement of pretreatment, and thus, it has been developed to be a prevalent technique for quantitative and qualitative analysis and well established in fields of agricultural engineering, food safety, environmental assessment, and medical and pharmaceutical science [9–12]. Consequently, NIR spectrometers are increasingly becoming a good choice for in situ measurements on the spots. However, in the quantitative prediction of fishmeal, there are many components other than protein (e.g., moisture, ash, and amino acids) which will raise major effects in the process of protein detection [13]. NIR fast detection is accompanied with noises from the nontarget components and interferences from the instable instrument.

NIR spectral response is detected at each resolvable wavelength to describe a sample to be tested. When hundreds of samples are tested, the NIR data are recorded as a spectral matrix as the incident NIR light that is preset in a certain frequency range is split into a bunch of wavelengths. But noting that not all of the wavelengths have a high signal-to-noise ratio, spectral features should be extracted to identify the informative wavelengths, and a calibration model should be established and optimized. In recent years, both theoretical evidence and experimental evidence show that variable selection is on critical demand to find out the spectral features corresponding to the target analyte component (e.g., the protein), so that the performance of the calibration model can be significantly improved [14, 15].

Many calibration methods are effectively applied to NIR quantitative analysis. Partial least squares (PLS) is a standard linear regression method commonly applied to NIR quantitative calibration in areas of agriculture, food, and biomedicine [16, 17]. The original form of PLS is to extract principal components both for the independent variables (i.e., the spectral wavelengths) and for the dependent variables (i.e., the contents of target components). It does not include any procedure of variable selection, and all variables are included in the original algorithm. Even though a linear subspace is built to suppress the influence of noise and the key factor of the latent variable is tunable for model optimization, the instinct noise interference of all variables is introduced into the regression procedure along with the target information [18, 19], and thus, the model predictive performance is limited and can hardly be improved.

Some refined methods related to variable selection were proposed, such as equivalent division of waveband [20], combined use of different subwavebands [21], moving window search of waveband [22], and competitive adaptive reweighted sampling of variables [23]. They are experimentally proved to be effective. Nevertheless, these methods mainly emphasize the selection of variables at their initial state (i.e., the wavelengths), or the extraction of principal component variables from the initial wavelengths. Seldom studies concern on a secondary implementable variable optimization, which is much necessary for the enhancement of variable selection and further for the improvement of model performance.

Iterative optimization algorithms can be considered for the secondary optimization of variable selection. Genetic algorithm (GA) is a kind of iterative optimization method that simulates the biological evolution in the sense of survival fitness [24]. It is on the basis of random global search with iterations for preset times. Selection, crossover, and mutation will be carried out in the process of iteration, so that the initial population of variables will evolve as new generations [25]. The genetic algorithm is well known and widely used for variable selection for spectroscopic analysis, embedded with the PLS or MLR regressions for quantitative calibration [26, 27].

In this paper, genetic algorithmic iteration was designed as the core process of the implementable optimization for variable selection in NIR quantitative analysis of fishmeal protein content. Firstly, the so-called informative wavebands were identified using the refined method grid search moving window (GSMW), where the calibration models were established by the classic PLS regression. Considering that protein is the only target analyte representing the variation of fishmeal nutrition supply, the search for latent variables in the PLS algorithm is preferable to be simplified as the analysis of latent principal component (LPC). Then, we are tending to launch a variable selection operation based on the principal component extraction combined with moving window technique (GSMW-LPC), where the genetic algorithm iteration is applied. The available LPCs extracted from each subwaveband will be further optimized by GA iteration, and parametric scaling mode is introduced for GA optimization. Consequently, the NIR calibration models were reestablished based on the GSMW-LPC-GA extracted variables, and the models were experimentally examined to improve the quantitative determination of protein contents in fishmeal samples. Hence, genetic iteration is regarded as an implementable optimization strategy for variable selection in NIR quantitative calibrations [28]. As different from previous studies, the design of GSMW-LPC-GA framework undertakes a hypercombined optimization of GSMW, LPC, and GA. Then, the calibration model can be optimized in joint optimal selection of MW parameters and the number of LPC and GA iterations. Especially in the GA process, the crossover rate, mutation rate, and the iteration times were designed for tuning, and a new subjection for stopping the GA iterations is identified. This strategy has the potential applicable prospectiveness for the nondestructive rapid detection of other analytes in fields of agriculture, environment, and animal husbandry.

## 2. Methods

### 2.1. The Parametric Scaling Genetic Algorithm for Variable Selection

In genetic algorithm, the trial individuals in each generation are encoded the gene according to its chromosomes using a specific language that guarantees a unique mapping from genotype to phenotype [29]. The phenotype results of the spectral calibration experiment are coded as genotype results so that its properties, components, and characteristic responses can be predicted. A loss function (also called fitness function or objectivity function) is defined to transform the genotype of an individual into a single quantitative value that represents the evolutionary performance of the individuals. The genetic algorithm is expressed as the following steps [30]:

  Step 1: a population is initially generated by randomly selecting a subset of variables (i.e., spectral wavelengths). The size of this population is preset. A flag is set for each wavelength in the full-scanning region. Each variable is successively marked with flags, where the flag is a binary marker equaling to 1 or 0. Flag 1 represents the corresponding variable is selected into the subset while Flag 0 represents the variable is not selected.
  Step 2: calibration models are established based on each variable subset, and thus, the model performance is validated and evaluated by computing the value of the loss function for the individuals (i.e., the variables) in the current population, in the way of cross-validation.
  Step 3: the calibration models are compared, and the next generation is produced by selecting a suitable variable subset that leads to improved performance with high prediction accuracy. The selected variable subsets are further modified by crossover or by mutation.
  Step 4: a modified variable subset is produced in one way by the crossover of the available variables between the selected variable subsets or in the other way by mutating the flags for each variable by small probability.
  Step 5: the selected variable subsets and the modified variable subsets are used to form a new variable population that should be taken as the inputs and return to step 2.

The steps 2–5 are repeated for iteration calculation, thus to generate several new populations. According to the modeling performance of cross-validation, the iteration will be stopped in one way when the model prediction error becomes stable or in the other way meeting the preset iteration times. The last generated population is regarded as the optimal informative variable subset.

The implementation of genetic algorithm depends on internal parameters such as population size, the probability of crossover (commonly set as 50%) and mutation operators (commonly set as 1.2%), and the stopping condition of iteration (commonly set as 500) [31]. The parametric scaling strategy should be applied to achieve the optimization of genetic evolution. The exploration of parametric optimization refers to the enhancement of the model prediction accuracy originated from the full-scanning variables regarding suitable genotype-phenotype coding and aiming to find the minimal value of the loss function.

### 2.2. The Grid Search Mode for Moving Window Optimization

Moving window (MW) is a simple and effective way to find the spectral informative features in a grid search optimization manner [32, 33]. Combined with the classical PLS regression, MW manages to extract spectral features in the study of chemometric algorithmic optimization for NIR rapid analysis, leading to appreciate prediction effects [34].

A window is initially defined by its size and its position. In particular, the size is annotated by the number of variables (*N*, i.e., spectral wavelengths) in the window, and a starting wavelength (*S*) represents the position of the window. When either *N* or *S* changes, the window moves through the full-scanning spectral range, with adjustable size. Thus, *N* and *S* are regarded as two parameters to determine a window (i.e., a subset of continuous wavelengths). A fix value of the combination of (*N*, *S*) precisely identifies a window, in which the variables will be utilized for establishing calibration models with some suitable quantitative regression algorithm.

With a grid search manner, the window parameters *N* and *S* are, respectively, set tunable in designated changing ranges. For any fixed value of *N*, *S* is set tunable from 1 to *P* − *N*+1 (here *P* represents the number of wavelengths in the full-scanning range); the calibration models are established, optimized, and evaluated. By comparing the model prediction results, the most optimal model corresponding to the fixed *N* can be determined, and simultaneously, the pairing value of *S* can also be found. On the counterpart, for any fixed value of *S*, *N* is set tunable from 1 to *P* − *S*+1; the calibration models are also compared to determine the most optimal model corresponding to the prevalued *S*, and the paring value of *N* is simultaneously observed. In this way, all possible windows are tested, and the most informative spectral waveband is identified.

The method of grid search moving window (GSMW) can be expressed in statistical formulae to identify the paring value of *N* (or *S*) for a fixed value of *S* (or *N*):

(1) $$\begin{array}{c} {\text{Model}}_{\text{opt}}\left(N_{\text{fixed}}\right)\cdot \text{window}=\left(N_{\text{fix}},\;\arg\left(\min\text{ ERR}\left(S_{\text{tunable}}\right)\left|N_{\text{fixed}}\right.\right)\right),\\ {\text{Model}}_{\text{opt}}\left(S_{\text{fixed}}\right)\cdot \text{window}=\left(\arg\left(\min\text{ ERR}\left(N_{\text{tunable}}\right)\left|S_{\text{fix}}\right.\right),S_{\text{fixed}}\right), \end{array}$$

where the term on the left of the equal sign represents the optimally found window (i.e., the most informative waveband), the term on the right is the optimally selected window size and position expressed in the form of (*N*, *S*), and ERR(·) is the loss function of the calibration model, which is usually defined as the model prediction error.

### 2.3. The Optimization Framework of GSMW-LPC-GA

NIR calibration models were established and optimized, where the informative wavebands were selected by using the grid search moving window operation. As the parameters of (*N*, *S*) were tunable, all possible applicable subwavebands were tested, and thus, several wavebands were identified with outputting appreciable modeling results. On this basis, a secondary optimization was carried out using the parametric scaling of GA as an implementable manner, and beforehand, the latent principal variable components are generated by the method of principal component analysis.

**Figure 1:**
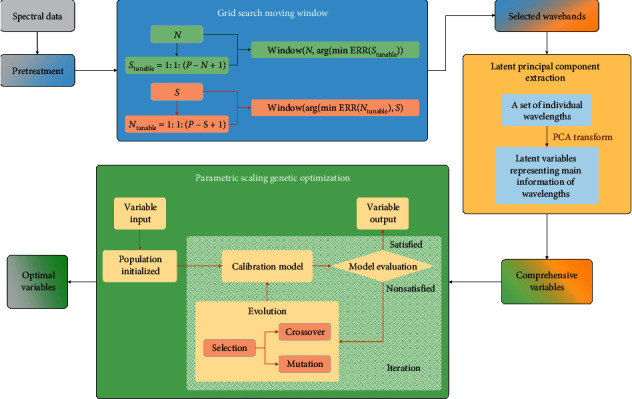
The flowchart of the optimization framework of GSMW-LPC-GA.

Figure 1 shows the flowchart of the GSMW-LPC-GA framework, and the algorithmic steps are expressed as follows:

 
*Framework Step 1*. Wavelengths in the full-scanning range of NIR spectroscopy were resolved as continuously changing variables. The spectral data are normalized and pretreated by the method of standard normal variate (SNV) [35].
 
*Framework Step 2*. The grid search moving window (GSMW) technique was applied to the full-length spectral range. In the way of screening all possible windows with applicable size and position, the optimal subwaveband can be identified, and some quasioptimal subwavebands (several appreciable parameter combinations of *N* and *S*) were found as well.
 
*Framework Step 3*. The wavelengths in the selected optimal and quasioptimal subwavebands were transformed to the principal variables using the principal component algorithm. Each new generated comprehensive variable of latent principal component (LPC) is expressed by a linear function of the initial wavelengths in the target subwaveband. The LPCs are sorted in a sequence with their descending information contribution, and significant LPCs can be obtained in front of the sequence. The LPCs should have been the optimal output variables for NIR predictions, but GA is designed in this framework as the implementable method for secondary optimization.
 
*Framework Step 4*. As for GA implementation, the designated significant LPCs are taken as the candidate individuals for GA inputs. The initial population is generated by randomly selecting a subset of the available LPCs and then goes through the steps of the parametric scaling GA iteration. The loss function is reasonably defined obeying the rule of minimizing the NIR prediction errors. Accompanied with the selection, crossover, and mutation processes, new generations of the population are repeatedly produced and cyclically refreshed. Thus, the secondary optimized informative variables are updated in loops until the iteration stops.

## 3. Data and Applications

### 3.1. Data Acquisition

Fishmeal samples were prepared as animal feeds. A total of 194 samples were collected for our experiment. 113 of them were acquired from Guangxi (an autonomous province in South China) and 81 were acquired from Vietnam. The samples were physically pretreated by air-drying, crushing, and sifting to particles with diameters no larger than 0.2 mm. The contents of protein were previously detected for each sample, using the conventional Kjeldahl method (Chinese National Standard: GB/T 6432-2018). The maximum and minimum values as well as the statistical average value and the standard deviation were recorded as 67.03, 53.17, 60.670, and 4.362 (wt.%), respectively.

**Figure 2:**
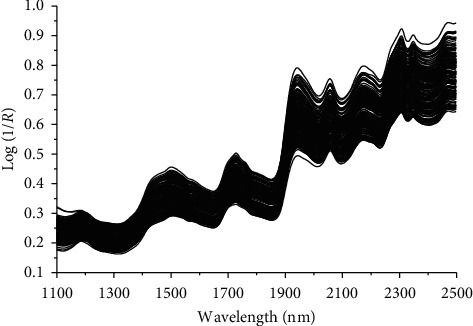
NIR reflectance spectra of 193 fishmeal samples.

The NIR spectral measurement was performed by using FOSS NIR Systems 5000 grating spectrometer (Foss NIRSystems Inc., Denmark) equipped with its diffuse reflectance accessory and a round sample cell. To reduce the systematic error, each sample was repeatedly detected for 5 times and the average spectrum was output as the spectrum of the sample. The temperature in the laboratory was controlled at 25 ± 1°C and the humidity was controlled at 70 ± 1% RH throughout the spectral scanning process. The NIR spectrum was measured over the wavenumber range of 1100–2500 nm with a resolution of 2 nm, so that 700 distincable wavelengths were identified as variables recording the spectral data. The spectra of 194 fishmeal samples are shown in Figure 2.

### 3.2. Sample Sets and Model Indicators

The proposed algorithmic framework of GSMW-LPC-GA was applied to variable selection for NIR calibration of fishmeal for the quantitative determination of protein content. For NIR calibrations, the fishmeal samples should be split into the modeling set and the testing set. As for modeling representativeness, the testing set was randomly picked in advance as including 54 samples, and the remaining samples were for modeling. Because the NIR calibration models need establishment and optimization, the modeling samples were further divided into the calibration part (90 samples) and the validation part (50 samples), where the method of sample set partitioning based on joint *x*-*y* distance [36] was applied. The maximum, minimum, and average values and the standard deviation for the calibration part, validation part, and testing set are listed in Table 1. The statistical data of all samples were also listed.

Quantitative indicators are needed to evaluate the calibration model. Root mean square error (RMSE) and correlation coefficients (*R*) are commonly used, which are defined as follows:

(2) $$\begin{array}{c} \text{RMSE}=\sqrt{\frac{\sum {\left(y_{i}-y_{i}^{\prime }\right)}^{2}}{n}},\\ R=\frac{\sum \left(y_{i}-y_{m}\right)\left(y_{i}^{\prime }-y_{m}^{\prime }\right)}{\sqrt{\sum {\left(y_{i}-y_{m}\right)}^{2}\sum {\left(y_{i}^{\prime }-y_{m}^{\prime }\right)}^{2}}}, \end{array}$$

where *y*_*i*_ and *y*_*i*_′ are the Kjeldahl-measured protein content and the NIR predictive value of the *i*-th fishmeal sample, *y*_*m*_ and *y*_*m*_′ are the mean value of {*y*_*i*_|*i*=1,2,…, *n*} and {*y*_*i*_′|*i*=1,2,…, *n*}, respectively, and *n* represents the number of target samples. Consequently, we denoted RMSEC/*R*_C_, RMSEV/*R*_V_, and RMSET/*R*_T_ as the corresponding signs of RMSE/R for the calibration samples, validation samples, and testing samples, respectively.

## 4. Results and Discussion

The optimization framework of GSMW-LPC-GA was applied to the quantitative analysis of fishmeal protein based on the NIR spectral matrix. In the framework, GSMW modeling method was used to select wavebands with high signal-to-noise ratio, which were regarded as the informative continuous wavelengths for further optimization. Next, latent variables were extracted from the initial continuous wavelengths, by using the latent principal component technique, so as to hold more information of the target with less designated variables. Then genetic algorithm was applied to promote the variable population evolution with steps of selection, crossover, and mutation. In the end, we managed to obtain some informative variables for the calibration of fishmeal protein, aiming to have a prospective improvement for the quality of NIR analysis.

### 4.1. Waveband Selection for the Partition of GSMW

GSMW is designed to use the grid search mode to optimize the way of parameter identification for the moving window algorithm flow. As is known that a specific window is determined by the position and its width (i.e., the number of variables in the window), the task of moving window optimization is to find the target parameter combinations of (*N*, *S*) that point to a solid window leading to optimal NIR prediction effect. In this work, we set *N* and *S* tunable, targeting 700 discrete initial scanning wavelengths. Considering that a model established on a large number of variables will increase the computational complexity, thus we design *N* valuing continuously when it is not over 100, and changing with a jumping step of 10 points when it is smaller than 300 and with a step of 20 when larger than 300 (i.e., *N* ∈ [1 : 1 : 100] ∪ [110 : 10 : 300] ∪ [320 : 20 : 700]). In this way, the value of *N* roughly went through some representative cases from 1 to 700 and also guaranteed a less computing load for acceptable operation with currently prevalent computers. On the other hand, the parametric value of S was set changing from 1 consecutively to 700 (i.e., *S* ∈ [1 : 1 : 700]). All applicable combinations of (*N*, *S*) should subject to the rule of *N*+*S* − 1 ≤ *P*, where *P* is equal to 700. Consequently, we totally tested 78, 790 possible windows.

**Figure 3:**
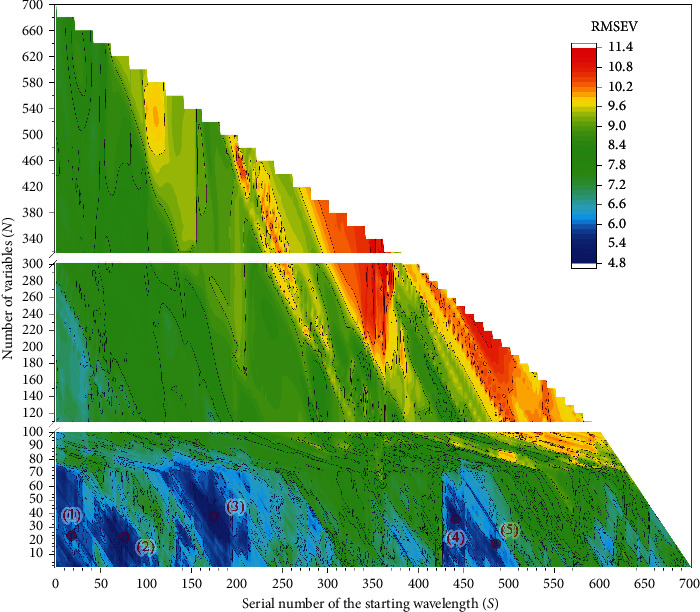
Contour plot of the validating results by the GSMW model.

**Figure 4:**
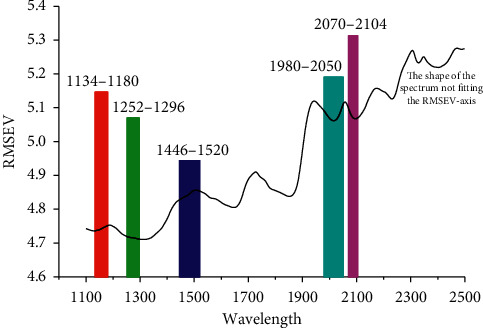
The locations of the 5 selected wavebands in the full spectral range.

PLS, the commonly used calibration method, was applied for each determinant window to estimate the functional contributions of the window wavelengths to the results of NIR calibrations. The calibration samples were used for model training and the validation samples for selecting the optimal model with a minimum value of RMSEV. The predictive RMSEV matrix is shown as a contour plot in Figure 3, corresponding to each fixed value of (*N*, *S*). Then, the most optimal window can be identified in Figure 3. Additionally, some well-performing models are expected to have further improvement in the next steps of the GSMW-LPC-GA framework. Thus, we finally determine to choose 5 optimal windows for further analysis (marked as (1), (2), (3), (4), and (5) in Figure 3). The prediction results as well as their operating parameters of the PLS models in these 5 windows are presented in Table 2, respectively. We found that the most optimal waveband was 1446–1520 nm, which includes 38 wavelengths/variables. Also we spotlight the selected 5 optimal wavebands in the full range (see Figure 4).

### 4.2. Genetic Optimization for LPCs in the Selected Wavebands

The selected 5 optimal wavebands were available for further optimization. The parameter scaling genetic algorithm was applied, with principal component analysis plugged in. The variables in each waveband were transformed to principal components (LPC) as latent variables for analysis. The number of LPCs is determined by the number of the original variables in the waveband. These latent components were sorted in descending contribution to its original data, which means that the latent variables in front of the queue contain more information than the variables in the back. Thus, the LPC transform projected the raw data into a reasonably refined variable space in which the target information is easier to observe. The original variables in windows (1), (2), (3), (4), and (5) were transformed into 24, 23, 38, 36, and 18 LPCs, respectively. All of the LPCs were used as the inputs for genetic optimization.

Genetic optimization is the vital implementation for variable selection embedded in the GSMW-LPC-GA framework. To make the GA procedure suitable for the selected input data, the population size was set fitting the inputs, and then the selection, crossover, and mutation were carried out. For parametric scaling tuning, the probability of crossover was set as 40%, 50%, and 60% for testing. The mutation operator was set testing the probabilities of 0.8%, 1.2%, and 1.5%. Then, the population automatically evolved in a loop iteration, and the loop was stopped in case of meeting each of the following two conditions, and the framework-optimized model was determined.

**Condition 1 .**

When reaching 500 times iteration, the loop was mandatorily stopped.

**Condition 2 .**

When there was no further change appearing in the loss function (i.e., the RMSEV) for consecutively 20 times iteration, the loop was adaptively stopped.

**Figure 5:**
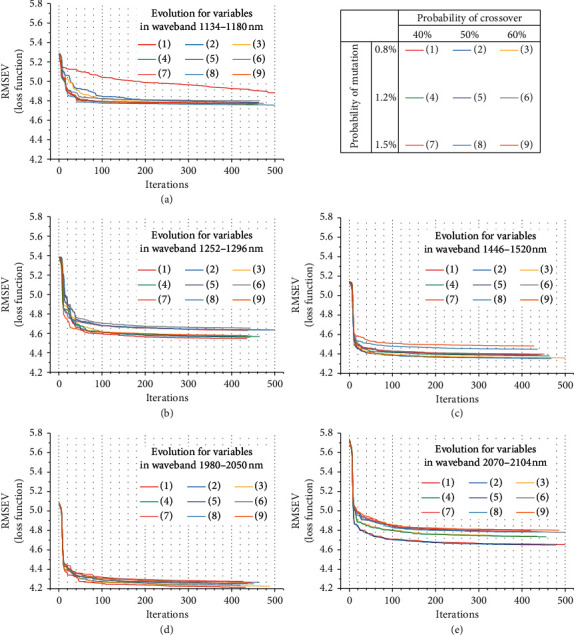
The optimizational effects for the principal variables in the 5 selected wavebands based on the parametric scaling GA iterations.

We tested the specific scaling parameter valuing of crossover and mutation in genetic algorithm for each of the 5 designated wavebands. The framework optimization results in the genetic evolution process are shown in Figure 5. It can be seen in Figure 5 that the evolution loop did not stop in some cases until 500 times iteration, and in other cases, the loop stopped before iteration reached 500 times. Finally, we were able to find the best results for each of the 5 selected wavebands by the genetic optimizational process. The secondary optimized RMSEV and the corresponding *R*_V_ are shown in Table 3, as well as their preferable GA parameters. We concluded from Table 3 that all of the data in the 5 selected wavebands were further optimized by GA and the most optimal result was observed in the waveband of 1980–2050 nm, whose evolution loop stopped at the 434 times iteration.

### 4.3. The Testing Results Based on the Optimal GSMW-LPC-GA Model

**Figure 6:**
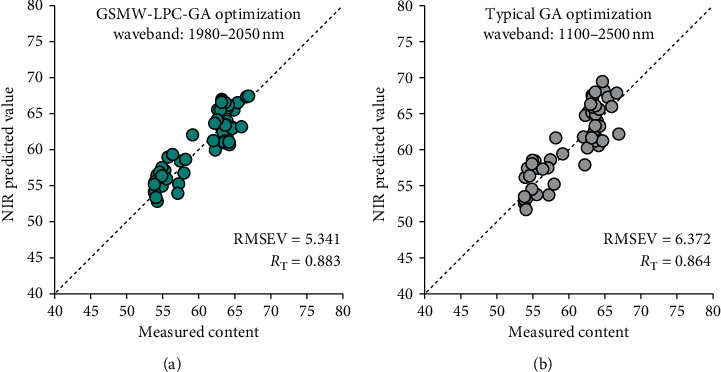
The PLS regression plot for test samples ((a) the proposed GSMW-LPC-GA framework and (b) the typical GA evolution).

As discussed above, the predictive performance of the GSMW-LPC-GA framework on the calibration and validation sample sets was satisfactory. The optimal model selected by the GSMW-LPC-GA framework was established on the waveband 1980–2050 nm (36 variables included), with genetic optimization at 40% crossover and 0.8% mutation. The evolution loop stopped at 434 times iteration. This framework optimal model outputs the prediction error RMSEV as 4.215 and the correlation *R*_V_ as 0.926. To evaluate the practical modeling effect of the framework, we took the waveband 1980–2050 nm as the target spectral data in the testing part, performed the principal component extraction, and applied the same GA parameters to estimate the modeling effect for the 53 testing samples, which were independent of the training part. The PLS regression plot is shown in Figure 6(a) demonstrating the testing results, where the RMSE_T_ and *R*_T_ were 5.341 and 0.883, respectively. As for comparison, the typical GA evolution method was used for establishing and optimizing the PLS calibration, validation, and testing processes. With common algorithmic settings, GA runs with 50% crossover, 1.2% mutation, and 500 times of iteration [31]. The GA-PLS testing results are shown as the regression plot in Figure 6(b). The comparative experiments show that the proposed GSMW-LPC-GA framework performs better than the typical GA evolution in the rapid NIR quantitative prediction of fishmeal protein.

## 5. Conclusions

NIR calibration model was established for the quantitative determination of protein content in fishmeal samples. A novel optimization framework of GSMW-LPC-GA was constructed for model optimization. Possible wavebands were tested for model enhancement by grid searching moving window parameters, in which the starting point was set continuously changing from 1 to the total number of wavelengths in the scanning range, and the window size was set in a specific design to balance the model representativeness and the computational complexity. Successively 5 optimal window wavebands were selected for further optimization by LPC feature extraction and GA iteration. Genetic algorithmic iteration was designed as the core process of implementable optimization. The variables/wavelengths in each of the 5 selected wavebands were transformed to LPCs as the input variables for GA iteration. In GA optimization, the population size was set obeying the dimensions of the input variable set. The probability of crossover and mutation was designed for parameter tuning. The control of iteration enlarged the extension of parametric scaling for generation evolutions. Finally, the model was secondarily optimized by the selection, crossover, and mutation operators in a refined interaction within the GSMW-LPC-GA framework.

The model prediction effects resulting from the GSMW-LPC-GA framework were experimentally proved better than the effect by simply adopting the moving window PLS model. In calibration-validation model training, the optimal GA probabilities for crossover and mutation were 40% and 0.8%, respectively, and the predictive RMSEV and RV were best as 4.215 and 0.926, respectively, at the waveband 1980–2050 nm. This is significantly better than the moving window output at the waveband 1446–1520 nm. For model evaluation, the best model generated from the GSMW-LPC-GA framework was further estimated by the testing sample set, which is previously chosen outside of the training samples. The testing results were not so good as the training results, but it was practically acceptable for some industrial rapid detection cases, with the RMSET and RT equaling to 5.341 and 0.883, respectively.

The experimental results demonstrate that the proposed GSMW-LPC-GA framework is suitable for NIR quantitative determination of fishmeal protein. It was able to produce better models, in relation to the full-spectrum model, with the advantage of selecting informative wavebands for target chemical analytes. In the framework, GA optimization eventually plays an important role in the improvement of the calibration model. Further studies include using the NIR technology to perform rapid detections on other targets in fields of animal husbandry, agriculture, and food science, and genetic optimization iteration is expected to be an implementable method providing an efficient strategy for analyzing some unknown changes and influence of various fertilizers.

## Figures and Tables

**Table 1:** The statistical data of fishmeal protein contents for the calibration part, validation part, and testing set.

	No. of samples	Maximum	Minimum	Average	Standard deviation
Calibration part	90	67.03	53.17	60.593	4.347
Validation part	50	66.19	53.86	59.174	4.412
Testing set	54	66.93	53.84	60.437	4.308
All samples	194	67.03	53.17	60.670	4.360

**Table 2:** The optimal model validating results corresponding to the 5 selected wavebands.

	*S*	*N*	Waveband (nm)	RMSEV (wt.%)	*R*_V_
(1)	18	24	1134–1180	5.146	0.874
(2)	77	23	1252–1296	5.071	0.884
(3)	174	38	1446–1520	4.944	0.905
(4)	441	36	1980–2050	5.190	0.892
(5)	486	18	2070–2104	5.312	0.908

**Table 3:** The optimal model validating results corresponding to the 5 selected wavebands.

Waveband (nm)	The optimal parameters for genetic evolution			RMSEV (wt.%)	*R*_V_
	Crossover (%)	Mutation (%)	Iteration times^#^		
1134–1180	50	1.5	500	4.755	0.906
1252–1296	40	0.8	437	4.546	0.897
1446–1520	50	0.8	469	4.353	0.918
1980–2050	40	0.8	434	4.215	0.926
2070–2104	50	0.8	484	4.649	0.912

^#^The iteration times represent when the optimal RMSEV was kept as a constant value for a cycle of 20 times iteration, or less than 20 in case the iteration had reached 500 times.

## Data Availability

The data used to support the findings of this study are available from the corresponding author upon request.
